# Side-Specific Movement Strategies During Maximal Arm Elevation in Individuals With Stroke: A Cross-Sectional Study

**DOI:** 10.7759/cureus.114946

**Published:** 2026-08-21

**Authors:** Yuji Osada, Tomo Osuka, Yuki Kuroda

**Affiliations:** 1 Department of Physical Therapy, Faculty of Health and Welfare, Tokushima Bunri University, Tokushima, JPN; 2 Department of Rehabilitation, Nakaizu Rehabilitation Center, Shizuoka, JPN

**Keywords:** arm elevation, biomechanics, compensatory movement, flexion synergy, kinematics, motion analysis, rehabilitation, stroke, upper extremity

## Abstract

Objectives

Compensatory movements of the paretic upper limb are commonly observed after stroke. Previous studies have mainly investigated tasks performed below shoulder height, such as reaching movements. However, activities of daily living also require arm elevation close to maximal range, which may impose greater mechanical demands and induce different compensatory strategies. This study aimed to determine differences in joint kinematics between paretic and nonparetic arm elevation at different points of the movement.

Methods

In this prospective cross-sectional study, 11 individuals with subacute hemiplegic stroke performed maximal forward elevation of the paretic and nonparetic upper limbs while seated in a standardized posture. Three-dimensional motion capture was used to analyze joint kinematics of the head, trunk, shoulder, and elbow. Joint angles were extracted at three points of the movement based on the percentage of maximal upper-arm segment flexion: early (10%), middle (50%), and peak (100%). Differences between paretic and nonparetic limb elevation were analyzed.

Results

During the early and middle points, no significant differences were observed in the compensatory kinematic variables other than the flexion angles used to characterize movement progression. However, at maximal elevation, elbow flexion was significantly greater during paretic limb elevation than during nonparetic limb elevation. In contrast, shoulder internal rotation was smaller during paretic limb elevation, and shoulder abduction did not differ significantly between conditions.

Conclusions

During maximal arm elevation, increased elbow flexion was one of the prominent kinematic differences observed between the paretic and nonparetic upper limbs, whereas excessive shoulder internal rotation was not observed. These findings highlight the importance of considering elbow flexion, in addition to shoulder rotation, when evaluating upper-limb movement in individuals with stroke.

## Introduction

Compensatory movements are frequently observed during activities of daily living in individuals with stroke, even in those who exhibit only minor clinical upper extremity impairment. Compared with healthy individuals, patients demonstrate increased forward trunk displacement and shoulder abduction during common tasks such as bringing a cup to the mouth, and these movement patterns have been reported to persist for more than one year after stroke onset [1].

In contrast, individuals with more severe motor impairment often show disuse of the paretic upper limb, resulting in characteristic postures such as elbow flexion and shoulder adduction and internal rotation. Such maladaptive postures may delay recovery of upper limb function and contribute to shoulder pain [2-4]. Given these compensatory movements and abnormal postures, it is widely considered important in clinical practice to promote the use of the paretic upper limb with movement patterns that are as close to normal as possible while minimizing excessive compensatory movements of the shoulder and trunk. For this reason, clinicians often focus on internal rotation of the paretic shoulder as a problematic movement and attempt to improve shoulder alignment by facilitating external rotation during rehabilitation [2,5-7].

Previous studies investigating compensatory movements of the paretic upper limb have primarily analyzed tasks performed below shoulder height, such as reaching movements [8]. However, activities of daily living involve a wide range of upper limb tasks, including reaching above the head. Tasks such as washing the body or retrieving objects from high shelves require shoulder motion approaching maximal elevation [9]. Compared with tasks performed on a tabletop, maximal arm elevation imposes greater mechanical demands and may therefore induce more pronounced compensatory movements. Despite this, few studies have investigated the kinematic characteristics of such high-demand tasks. Analyzing compensatory movements under these conditions may therefore provide useful insights into upper limb motor control in individuals with stroke. Because maximal arm elevation involves substantial changes not only in shoulder flexion but also in rotational motion, understanding the role of shoulder internal and external rotation during this task is particularly important.

Contrary to the clinical assumption that excessive internal rotation occurs as a compensatory movement during use of the paretic upper limb, kinematic studies of shoulder rotation during arm elevation have reported somewhat different findings. Shoulder abduction is typically accompanied by external rotation of the humerus. In contrast, during shoulder flexion, the greater tuberosity passes through the anterior portion of the coracoacromial arch to avoid impingement, and the humerus gradually internally rotates as flexion progresses [10]. Furthermore, the glenohumeral joint in individuals with stroke has been reported to show increasing internal rotation after approximately 70° of shoulder flexion during arm elevation [11]. The overall rotational pattern appears similar between the two groups, although the magnitude of internal rotation tends to be slightly greater than that observed in healthy individuals [11]. Additionally, no significant difference has been reported in rotational angles at maximal forward elevation of the upper limb, while the range of external rotation at maximal abduction has been shown to be reduced in individuals with stroke compared with healthy individuals [11]. These findings suggest that internal rotation during arm elevation is not necessarily an abnormal movement, and its clinical significance remains insufficiently understood.

More than 80% of individuals with stroke exhibit complex compensatory movements involving the wrist, shoulder, and trunk during object manipulation [12]. Moreover, compensatory trunk movements such as trunk extension, contralateral lateral flexion, and contralateral rotation have been observed regardless of movement speed during arm elevation [13]. Multiple compensatory movements may occur simultaneously during maximal arm elevation because such conditions require a larger range of motion than tasks performed on a tabletop. However, it remains unclear when these compensatory movements begin and which joints are primarily involved during the movement.

Therefore, the purpose of this study was to clarify when and in which joints compensatory movements occur during maximal elevation of the paretic upper limb in individuals with stroke by comparing this movement with elevation of the nonparetic limb in the sagittal plane. In particular, we examined whether compensatory movements are characterized primarily by excessive shoulder internal rotation or whether they are accompanied by other compensatory strategies, such as trunk extension and contralateral lateral flexion. We hypothesized that during forward elevation of the paretic upper limb, multiple compensatory movements, including excessive shoulder internal rotation as well as trunk extension and contralateral lateral flexion, would appear early in the movement.

## Materials and methods

Participants

This prospective cross-sectional study was conducted at Nakaizu Rehabilitation Center from September 1, 2015, to February 28, 2016. The inclusion criteria for this study were as follows: patients admitted to a convalescent rehabilitation ward with subacute stroke, presence of unilateral upper limb motor paralysis, first-ever stroke, no marked limitation in shoulder joint range of motion, and the ability to maintain a stable seated position. Patients were excluded if they had orthopedic disorders involving the spine or shoulder, severe shoulder pain during arm elevation, or severe upper-limb motor impairment (Brunnstrom Recovery Stage; BRS < III). Eleven individuals with hemiplegia (mean age, 60.8 ± 10.7 years) participated in this study. Because this was an exploratory study using an available clinical sample, an a priori sample-size calculation was not performed. As part of the ethical considerations, all participants received both written and verbal explanations of the study and provided written informed consent prior to participation. This study was conducted after approval had been obtained from the ethics committees of the Tokushima Bunri University (approval no. R7-38) and the Nakaizu Rehabilitation Center (approval no. 15001).

Clinical assessment

Upper-limb motor recovery was evaluated using the Brunnstrom Recovery Stage (BRS), which classifies motor recovery into six stages ranging from flaccid paralysis (Stage I) to near-normal isolated movement (Stage VI) [14]. Upper-limb motor function was also assessed using the Fugl-Meyer Assessment (FMA). The upper-extremity section of the FMA consists of 33 items scored on a three-point ordinal scale (0-2), yielding a maximum score of 66, with higher scores indicating better motor function [15,16]. Both assessments were performed as part of routine clinical practice by the participants' treating physical therapists during the same period as the motion analysis, and the corresponding BRS and FMA scores were extracted from the medical records for analysis.

Procedure

**Figure 1:**
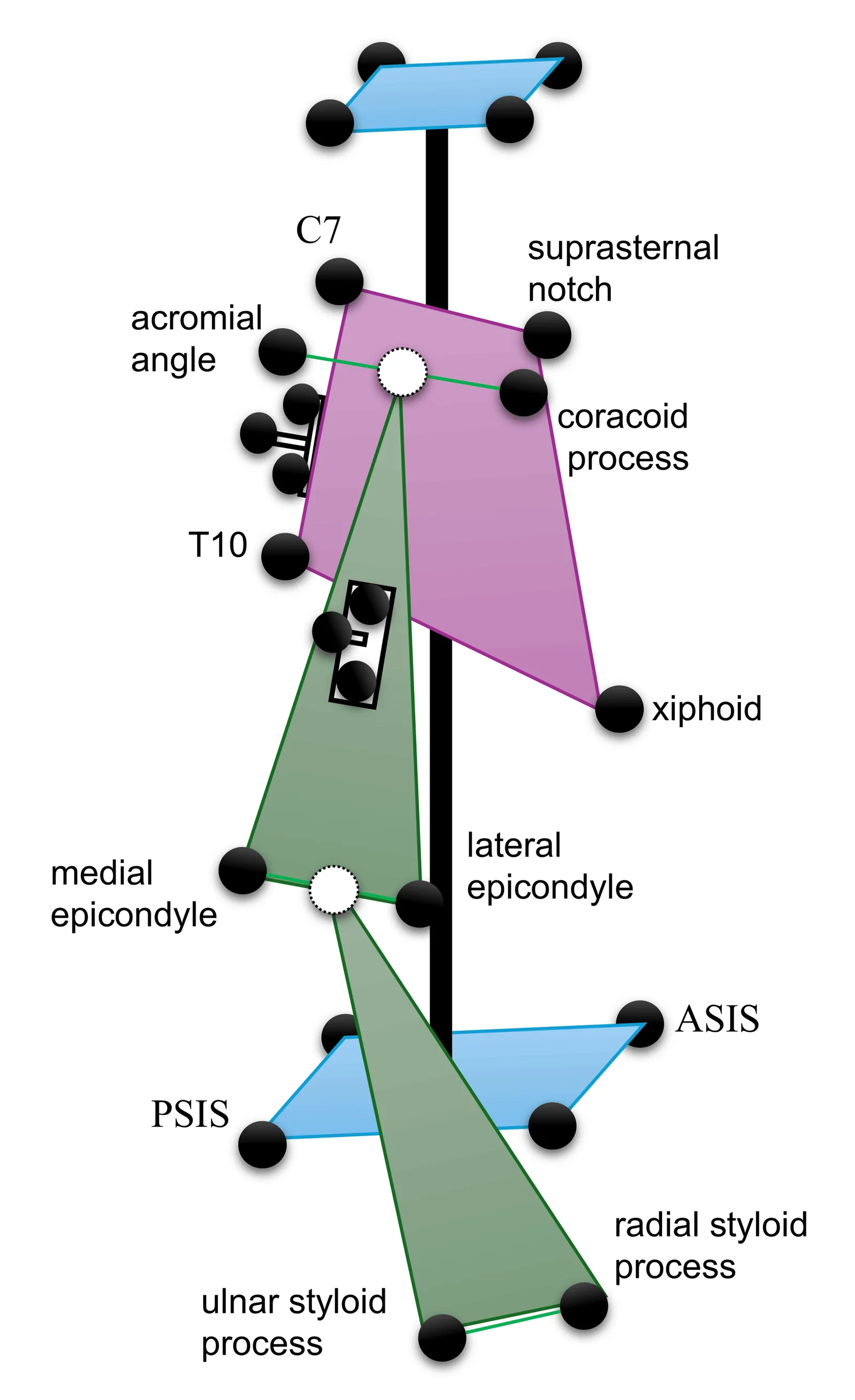
Schematic representation of reflective marker placement and segment definitions for motion analysis Black circles indicate reflective markers, whereas white circles indicate calculated joint centers. The shoulder joint center was defined as the midpoint between the acromial angle and coracoid process, and the elbow joint center was defined as the midpoint between the medial and lateral epicondyles. Blue quadrilaterals represent the head and pelvic segments, purple represents the upper trunk segment, and green represents the upper-arm and forearm segments. Three-marker jigs attached to the upper arm and trunk are shown as small black rectangular marker clusters. C7, seventh cervical vertebra; T10, tenth thoracic vertebra; ASIS, anterior superior iliac spine; PSIS, posterior superior iliac spine.

The experimental task consisted of maximal arm forward elevation. Participants were seated on a 40-cm-high platform with their hands resting on their knees and were instructed to elevate each arm (paretic and non-paretic) as high as possible, three times. The movement was performed at a controlled speed: participants raised the arm to the maximum possible height over 3 seconds. The task was recorded using a three-dimensional motion capture system consisting of eight infrared cameras (Vicon Nexus; Vicon, Oxford, UK). To standardize each joint angle, a static sitting trial was recorded while the participant’s posture was passively adjusted to shoulder flexion, abduction, and external rotation of 0°, with the elbow flexed at 90°. Participants wore minimal clothing, and reflective markers were attached directly to the skin whenever possible. For female participants, a compression suit was worn, and the reflective markers were attached over the suit. A total of 24 reflective markers were placed on anatomical landmarks across the body (Figure 1), with an additional three-marker jig attached to each upper arm and the trunk.

The upper-arm segment was defined using three markers placed at the shoulder joint center (midpoint between the coracoid process and the acromial angle), the medial epicondyle, and the lateral epicondyle [17]. A three-marker jig was attached at the midpoint of the line connecting the acromion and lateral epicondyle of each upper arm and was used to track upper-arm motion when the medial or lateral epicondyle marker became temporarily obscured during movement. In such cases, the jig trajectory was used to reconstruct the missing epicondyle marker trajectory. The forearm segment was defined using the midpoint between the medial and lateral epicondyles of the humerus and markers placed on the radial and ulnar styloid processes. The upper trunk segment was defined by four markers placed at the seventh cervical vertebra (C7), the tenth thoracic vertebra (T10), the suprasternal notch, and the xiphoid process. An additional three-marker jig was attached to the trunk between C7 and T10 and was similarly used to reconstruct missing trunk marker trajectories when necessary. To define the head segment, participants wore a headband with four embedded reflective markers. The pelvic segment was defined using four markers placed on the bilateral anterior superior iliac spines and posterior superior iliac spines. The pelvic coordinate system was constructed using the bilateral anterior superior iliac spine markers and the midpoint of the bilateral posterior superior iliac spine markers [17].

The sampling frequency was set at 100 Hz, and the data were filtered using a 6-Hz low-pass filter [18]. Kinematic data were calculated using three-dimensional motion analysis software (Visual3D; HAS-Motion, Inc., Kingston, Ontario, Canada). Joint angles were calculated using an X-Y-Z Cardan angle sequence based on the local coordinate systems defined for each segment, with rotations about the X-, Y-, and Z-axes representing flexion/extension, abduction/adduction, and axial rotation, respectively. This rotation sequence was selected to facilitate the clinical interpretation of flexion/extension, abduction/adduction, and axial rotation components [19]. Although Cardan angles are theoretically susceptible to gimbal lock near extreme joint positions, previous studies have reported this potential issue when shoulder flexion approaches 0° or 180° [20]. In the present study, neither the shoulder nor elbow joint angles approached configurations associated with gimbal lock.

The following kinematic variables were extracted: head angle relative to the global coordinate system, thoracic angle relative to the pelvis (trunk angle), upper-arm segment angle relative to the global coordinate system, upper-arm segment angle relative to the upper trunk (shoulder joint angle), and forearm angle relative to the upper-arm segment (elbow flexion and pronation/supination angles). Shoulder internal rotation was a principal kinematic variable of interest based on the study hypothesis, while trunk movements and other joint kinematics, including elbow motion, were examined to characterize accompanying compensatory movement patterns. No single primary outcome was prespecified because this exploratory study was designed to characterize compensatory movements across multiple body segments. For each trial, the elevation movement was individually normalized from movement onset (0%) to the peak upper-arm segment flexion angle (100%). Kinematic values were then extracted at the 10%, 50%, and 100% points of each normalized trial, and the values obtained from the three trials were averaged for each condition and used for statistical analysis. Because normalization was performed relative to the maximum upper-arm segment flexion achieved in each trial, the same normalized point did not necessarily correspond to the same absolute elevation angle between the paretic and non-paretic conditions. The 10%, 50%, and 100% points of normalized upper-arm segment flexion were selected to represent early, middle, and maximal elevation, respectively. Previous studies have demonstrated that muscle activity and trunk control vary across the progression of arm elevation, with distinct changes observed during the early and later stages of the movement [13]. Therefore, these three points were selected to characterize how kinematic differences between the paretic and non-paretic conditions emerged as arm elevation progressed.

Statistical analysis

To examine differences in posture between forward elevation of the paretic and non-paretic limbs, the normality of the paired differences for each variable was assessed using the Shapiro-Wilk test. Normally distributed variables were compared using paired t-tests, whereas non-normally distributed variables were compared using the Wilcoxon signed-rank test. For normally distributed variables, paired mean differences with 95% confidence intervals (CIs) were calculated. For non-normally distributed variables, the median of the paired differences with bootstrap 95% CIs was calculated. To control the false discovery rate associated with multiple comparisons, p-values were adjusted using the Benjamini-Hochberg method. The significance level was set at α = 0.05. Effect sizes were calculated using Cohen’s d for parametric comparisons and effect size r for non-parametric comparisons. All statistical analyses were performed using Python version 3.12.4 (Python Software Foundation), with the SciPy and statsmodels libraries.

## Results

The demographic and clinical characteristics of the participants are summarized in Table 1.

**Table 1:** Participant characteristics Continuous variables are presented as mean ± standard deviation (SD) with ranges (minimum–maximum). Categorical variables are presented as n (%). BMI, body mass index; BRS, Brunnstrom Recovery Stage; FMA, Fugl-Meyer Assessment.

Characteristic	Value
Number of participants	11
Age, years	60.8 ± 10.7 (40-75)
Time since stroke, days	111.2 ± 47.1 (50-176)
Sex	
Female	1 (9.1%)
Male	10 (90.9%)
Paretic side	
Left	6 (54.5%)
Right	5 (45.5%)
Type of stroke	
Hemorrhagic	6 (54.5%)
Ischemic	5 (45.5%)
BMI, kg/m^2^	23.4 ± 3.4 (20.8-33.1)
BRS, upper extremity	
III	3 (27.3%)
IV	2 (18.2%)
V	6 (54.5%)
FMA, upper extremity subscore	51.8 ± 16.0 (21-65)

**Figure 2:**
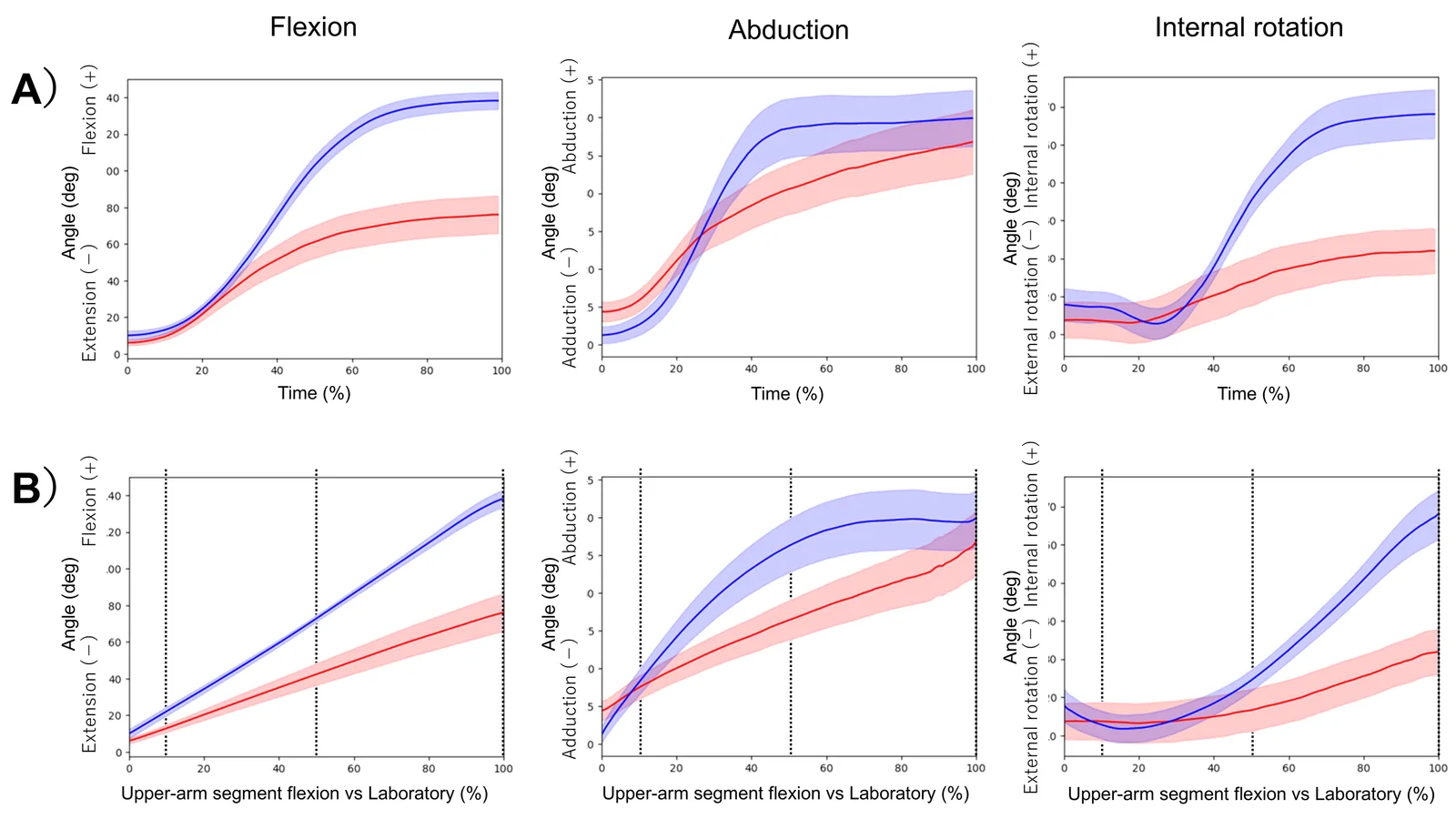
Comparison of shoulder joint angle changes normalized to time and upper-arm segment flexion (n = 11) The graphs show changes in shoulder flexion, abduction, and internal rotation relative to the thorax. Trajectories of the paretic upper limb are shown as solid red lines (mean) with shaded areas representing the standard error of the mean (SEM), whereas those of the non-paretic upper limb are shown as solid blue lines with shaded areas representing SEM. Positive values indicate shoulder flexion, abduction, and internal rotation. The upper panels (A) show data normalized to time from movement onset (0%) to the completion of the movement (100%). The lower panels (B) show data normalized to upper-arm segment flexion from movement onset (0%) to maximal flexion (100%). In panel B, upper-arm segment flexion was defined relative to the laboratory coordinate system ("Laboratory"), rather than relative to the thorax. Vertical dashed lines indicate 10%, 50%, and 100% of normalized upper-arm segment flexion.

This study compared compensatory movements between elevation of the paretic and non-paretic upper limbs during the early, middle, and peak points of maximal arm forward elevation (Figure 2). 

Table 2 presents the differences in joint angles between the paretic and non-paretic conditions at each point.

**Table 2:** Kinematic differences between the paretic and non-paretic sides during upper-limb elevation (n = 11) Because the points were defined relative to the maximal upper-arm segment flexion achieved in each trial, between-condition differences in absolute upper-arm segment and shoulder flexion angles partly reflect differences in maximal elevation range. Elevation point (%) represents normalized upper-arm segment flexion point (10%: early point, 50%: mid point, 100%: peak flexion). Sign convention: Positive angle values indicate extension for the head and trunk, and flexion, abduction, internal rotation, and pronation for the upper-limb joints. Positive values for head and trunk lateral bending and rotation represent movements toward the arm elevation side, whereas negative values indicate the opposite directions for the corresponding movements. Values are presented as mean ± standard deviation (SD) or median (lower quartile–upper quartile). For parametrically analyzed variables, differences are presented as paired mean differences with 95% confidence intervals (CIs). For non-parametrically analyzed variables, differences are presented as median paired differences with percentile bootstrap 95% CIs based on 2,000 resamples. Angular variables are expressed in degrees (°). False discovery rate (FDR) correction was applied using the Benjamini-Hochberg method. ^a^Cohen’s d was used for parametric tests, and effect size r for non-parametric tests. ^b^Wilcoxon signed-rank test. ^c^Paired t-test.

Elevation point	Variable	Paretic side	Non-paretic side	Difference (95% CI)	Test statistic	Effect size^a^	p-value (FDR)
Early (10%)	Head angle (extension/flexion)^b^	-25.5 (-27.2 to -21.4)	-22.9 (-27.5 to -17.7)	0.0 (-6.5 to 3.0)	W = 28	0.834	0.134
	Head angle (lateral bending)^c^	-0.3 ± 6.7	-1.9 ± 5.7	1.6 (-6.5 to 9.7)	t = 0.44	0.131	0.807
	Head angle (rotation)^c^	-2.9 ± 10.2	6.3 ± 8.7	-9.2 (-21.7 to 3.3)	t = -1.63	-0.493	0.386
	Trunk angle (extension/flexion)^c^	-0.6 ± 3.9	-0.3 ± 4.1	-0.2 (-2.2 to 1.7)	t = -0.28	-0.083	0.860
	Trunk angle (lateral bending)^c^	0.3 ± 4.2	-2.7 ± 3.2	2.9 (-1.7 to 7.6)	t = 1.40	0.422	0.462
	Trunk angle (rotation)^c^	1.6 ± 8.3	-3.1 ± 7.4	4.7 (-5.7 to 15.2)	t = 1.01	0.304	0.606
	Upper arm segment angle (flexion)^c^	11.7 ± 6.5	26.3 ± 7.4	-14.6 (-21.8 to -7.3)	t = -4.45	-1.343	0.009
	Shoulder joint angle (flexion)^c^	13.4 ± 6.7	24.7 ± 9.6	-11.3 (-20.9 to -1.6)	t = -2.61	-0.787	0.117
	Shoulder joint angle (abduction)^c^	18.7 ± 4.8	20.5 ± 9.4	-1.8 (-7.6 to 3.9)	t = -0.71	-0.214	0.724
	Shoulder joint angle (internal rotation)^c^	14.0 ± 15.7	11.9 ± 13.6	2.1 (-13.8 to 18.1)	t = 0.30	0.090	0.860
	Elbow angle (flexion)^c^	55.6 ± 14.7	51.7 ± 18.2	3.9 (-10.6 to 18.3)	t = 0.60	0.180	0.779
	Elbow angle (pronation/supination)^c^	14.7 ± 19.0	17.8 ± 19.5	-3.0 (-15.6 to 9.5)	t = -0.54	-0.163	0.801
Mid (50%)	Head angle (extension/flexion)^c^	-29.4 ± 7.5	-26.2 ± 6.1	-3.2 (-7.4 to 1.0)	t = -1.68	-0.505	0.386
	Head angle (lateral bending)^c^	-2.3 ± 8.1	-3.5 ± 6.1	1.2 (-8.0 to 10.5)	t = 0.30	0.091	0.860
	Head angle (rotation)^c^	-2.2 ± 9.6	6.4 ± 8.7	-8.6 (-20.5 to 3.3)	t = -1.61	-0.484	0.386
	Trunk angle (extension/flexion)^c^	3.1 ± 4.6	1.4 ± 4.1	1.7 (-1.1 to 4.5)	t = 1.33	0.402	0.476
	Trunk angle (lateral bending) ^b^	1.2 (-1.7 to 1.9)	-4.7 (-6.8 to -1.7)	5.5 (-3.8 to 8.6)	W = 20	0.589	0.348
	Trunk angle (rotation)^c^	1.6 ± 7.6	-3.7 ± 7.4	5.3 (-4.7 to 15.3)	t = 1.18	0.355	0.533
	Upper arm segment angle (flexion)^c^	46.5 ± 21.6	79.1 ± 7.5	-32.6 (-47.3 to -17.9)	t = -4.94	-1.488	0.008
	Shoulder joint angle (flexion)^c^	43.3 ± 20.2	70.9 ± 9.9	-27.5 (-43.4 to -11.7)	t = -3.86	-1.165	0.019
	Shoulder joint angle (abduction)^c^	27.5 ± 8.3	39.3 ± 17.5	-11.8 (-24.9 to 1.2)	t = -2.02	-0.610	0.283
	Shoulder joint angle (internal rotation)^c^	17.5 ± 18.3	21.0 ± 10.2	-3.4 (-20.1 to 13.3)	t = -0.46	-0.138	0.807
	Elbow angle (flexion)^c^	62.6 ± 17.3	57.6 ± 19.2	5.0 (-11.0 to 20.9)	t = 0.70	0.210	0.724
	Elbow angle (pronation/supination)^c^	9.0 ± 17.0	13.0 ± 19.0	-3.9 (-14.3 to 6.4)	t = -0.85	-0.256	0.681
Peak (100%)	Head angle (extension/flexion)^b^	-34.0 (-38.4 to -31.7)	-37.1 (-41.2 to -33.6)	2.4 (-4.1 to 6.4)	W = 29	0.834	0.107
	Head angle (lateral bending)^c^	-3.5 ± 9.4	-7.4 ± 6.4	4.0 (-6.2 to 14.1)	t = 0.86	0.261	0.681
	Head angle (rotation)^c^	-4.4 ± 10.2	2.2 ± 10.5	-6.5 (-19.3 to 6.3)	t = -1.13	-0.342	0.536
	Trunk angle (extension/flexion)^c^	8.5 ± 6.5	7.5 ± 5.3	0.9 (-3.3 to 5.2)	t = 0.50	0.150	0.807
	Trunk angle (lateral bending)^c^	-2.7 ± 5.7	-6.6 ± 4.5	3.9 (-2.2 to 10.1)	t = 1.42	0.428	0.462
	Trunk angle (rotation)^c^	4.2 ± 6.0	-2.7 ± 7.4	6.9 (-1.9 to 15.7)	t = 1.74	0.525	0.386
	Upper arm segment angle (flexion)^c^	89.3 ± 41.1	146.8 ± 12.4	-57.5 (-85.2 to -29.8)	t = -4.63	-1.395	0.008
	Shoulder joint angle (flexion)^c^	77.0 ± 34.5	139.2 ± 13.5	-62.2 (-85.9 to -38.5)	t = -5.85	-1.765	0.006
	Shoulder joint angle (abduction)^c^	38.0 ± 14.1	39.2 ± 12.9	-1.2 (-16.1 to 13.6)	t = -0.19	-0.056	0.890
	Shoulder joint angle (internal rotation)^c^	33.5 ± 19.7	69.2 ± 21.8	-35.8 (-52.8 to -18.7)	t = -4.68	-1.410	0.008
	Elbow angle (flexion)^c^	71.9 ± 21.2	47.5 ± 17.9	24.4 (9.3 to 39.5)	t = 3.59	1.083	0.025
	Elbow angle (pronation/supination)^c^	2.6 ± 17.0	3.4 ± 19.3	-0.8 (-11.4 to 9.8)	t = -0.17	-0.053	0.890

At the early (10% of upper-arm segment flexion) and middle (50% of upper-arm segment flexion) points of arm elevation, the upper-arm segment flexion angle during elevation of the non-paretic limb was approximately 15° and 33° greater, respectively, than that during elevation of the paretic limb (Figure 2B). Because each trial was normalized to its peak upper-arm segment flexion, the absolute upper-arm segment and shoulder flexion angles were smaller during paretic limb elevation at the early and middle points. These differences primarily reflected the smaller maximal elevation range of the paretic limb and were not interpreted as compensatory movements. No significant differences were observed in the other head, trunk, shoulder, or elbow variables at these points.

**Figure 3:**
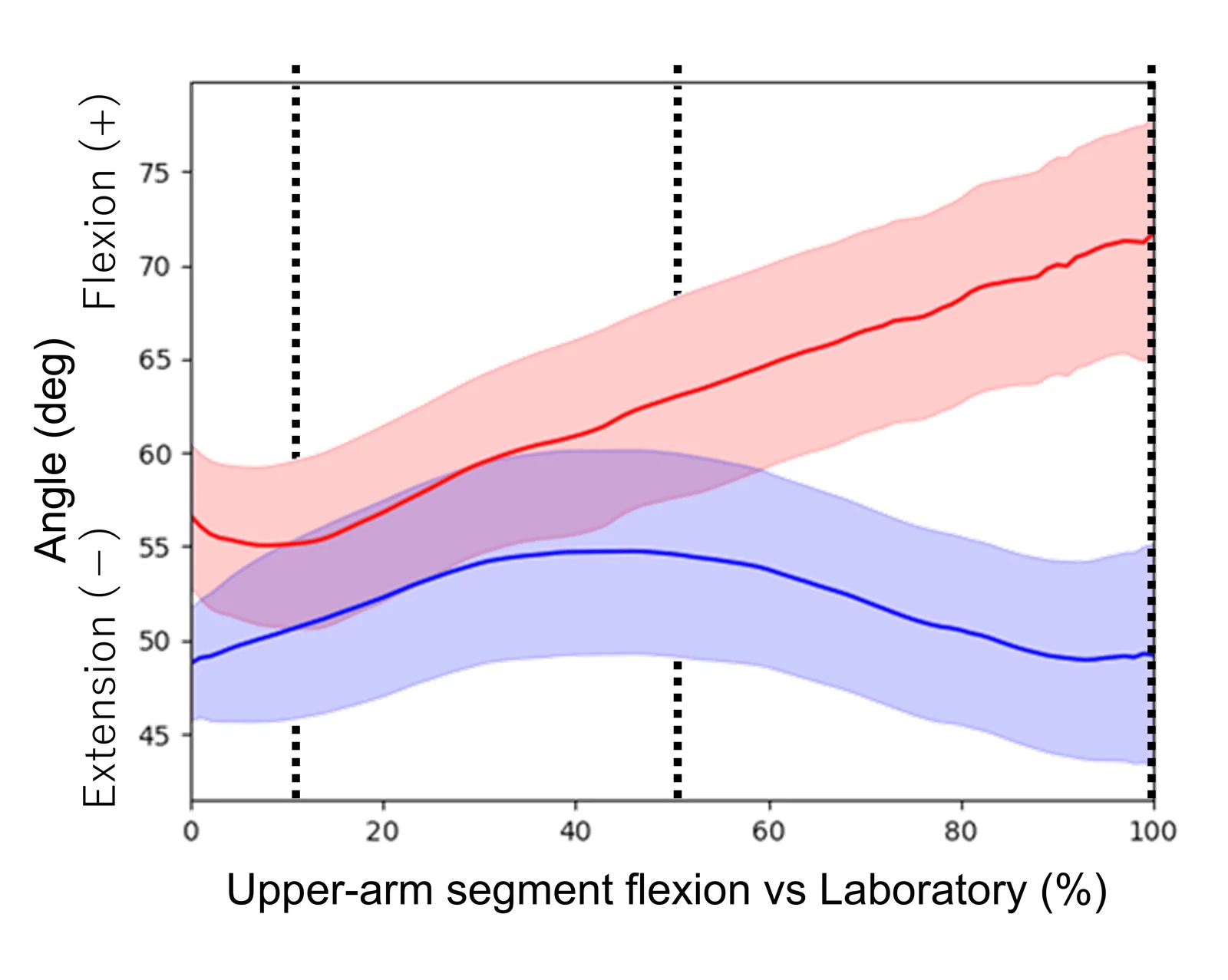
Comparison of elbow flexion angle changes normalized to upper-arm segment flexion (n = 11) The graphs show changes in elbow flexion angle. Trajectories of the paretic upper limb are shown as solid red lines (mean) with shaded areas representing the standard error of the mean (SEM), whereas those of the non-paretic upper limb are shown as solid blue lines with shaded areas representing SEM. Positive values indicate elbow flexion. The data were normalized to upper-arm segment flexion from movement onset (0%) to maximal flexion (100%). Upper-arm segment flexion was defined relative to the laboratory coordinate system ("Laboratory"). Vertical dashed lines indicate 10%, 50%, and 100% of normalized upper-arm segment flexion.

However, at maximal elevation (100% of upper-arm segment flexion), significant differences were also observed in shoulder internal rotation and elbow flexion (Figures 2, 3).

During elevation of the paretic limb, elbow flexion increased by approximately 24° compared with that during elevation of the non-paretic limb (p = 0.025). In contrast, shoulder internal rotation was smaller during elevation of the paretic limb and was approximately half of that observed during elevation of the non-paretic limb (p = 0.008) (Figure 2).

## Discussion

To clarify compensatory movements during paretic arm elevation, this study compared joint kinematics during forward elevation of the paretic and non-paretic upper limbs. We hypothesized that shoulder abduction and internal rotation would appear early during elevation of the paretic limb. However, the present results showed no substantial postural differences between the two conditions up to the middle point of elevation. Clear compensatory movements were observed only at maximal elevation. Even at that point, shoulder abduction did not differ significantly between conditions, and shoulder internal rotation was not excessive during elevation of the paretic limb; rather, internal rotation was significantly smaller than during elevation of the non-paretic limb. Instead, increased elbow flexion was one of the prominent kinematic differences observed at maximal elevation.

Meaningful between-condition differences in compensatory movements emerged only at the final elevation position; the earlier differences in upper-arm and shoulder flexion primarily reflected the normalization procedure and the smaller maximal elevation range of the paretic limb. Maximal arm forward elevation requires not only maximal shoulder flexion but also coordinated movements of the scapula and trunk [13]. Because no significant differences in the other kinematic variables were observed up to 50% of elevation, reaching this level may not require excessive effort for individuals with stroke. However, as the arm approaches the maximal elevation position and the available range of motion becomes limited, compensatory movements may become more evident.

A prominent kinematic difference observed during maximal elevation of the paretic limb was increased elbow flexion. The mean difference in elbow flexion between conditions was approximately 24°, indicating that the elbow was flexed about 1.5 times more during paretic limb elevation than during non-paretic limb elevation. One possible explanation for the increased elbow flexion is the influence of flexion synergy; however, flexion synergy was not directly quantified in the present study. Nearly half of the individuals in this study had a Brunnstrom Recovery Stage of 4 or lower in the paretic upper limb, indicating limited ability to perform isolated movements. Because the severity of motor impairment has been reported to be positively associated with the appearance of flexion synergy during arm elevation [21], the presence of moderate motor impairment in the participants may have contributed to this finding. In addition, elbow flexion shortens the effective length of the upper limb and may reduce the external moment acting on the shoulder joint, thereby allowing the arm to be elevated to a higher position.

Another notable finding was that shoulder internal rotation did not appear as a major compensatory movement. One possible explanation is that internal rotation is a normal accompanying motion during sagittal-plane arm elevation (i.e., arm forward elevation). Previous studies have reported that the humerus internally rotates relative to the thorax during forward elevation of the arm in healthy individuals [10,22]. In the present study, shoulder internal rotation during elevation of the non-paretic limb occurred at approximately half the magnitude of shoulder flexion, indicating a roughly 2:1 relationship between flexion and internal rotation. A similar relationship was observed during elevation of the paretic limb. Because the maximal shoulder flexion angle during non-paretic limb elevation was approximately 1.5 times greater than that during paretic limb elevation, the smaller internal rotation angle observed during paretic limb elevation may simply reflect the smaller flexion angle. However, internal rotation was relatively large compared with the flexion angle at the early (10%) point of paretic limb elevation. Previous studies have reported delayed muscle onset during the initial phase of upper limb movement in individuals with stroke, with excessive activation of the anterior deltoid and biceps brachii occurring as a compensatory response [23]. Such instability of the forearm segment during the early phase of arm elevation may have resulted in passive internal rotation at the shoulder. Contrary to common clinical assumptions that the paretic upper limb tends to exhibit excessive internal rotation during movement, the present findings showed that internal rotation at maximal elevation was actually smaller than that of the non-paretic limb, which represents an interesting and previously unreported observation.

From a clinical perspective, the present results do not support the need to primarily correct excessive shoulder internal rotation during maximal arm elevation of the paretic limb. In addition, shoulder abduction and trunk movements did not differ significantly from those observed during non-paretic limb elevation. Therefore, no consistent trunk compensatory pattern, as commonly described during paretic arm elevation in clinical settings, was identified in the present participants, possibly due to the large variability in trunk movements reflecting diverse compensatory strategies across individuals. Instead, the present findings suggest that elbow flexion should also be considered when evaluating upper-limb movement during forward elevation. Furthermore, similar compensatory movements may also occur during elevation of the non-paretic limb. At maximal elevation, cervical flexion and trunk extension were observed during elevation of both limbs. However, because healthy controls were not included, these movements cannot be definitively interpreted as compensatory strategies. These findings indicate that clinical evaluation should consider not only shoulder internal rotation of the paretic limb but also elbow motion and movements occurring during elevation of the non-paretic limb.

This study has some limitations. First, the sample size was small, and no a priori sample size calculation was performed. In addition, because a healthy control group was not included, the observed differences between the paretic and non-paretic limbs cannot be interpreted as movement patterns specific to individuals with stroke. Second, participants with severe motor impairment who were unable to perform the elevation task were not included, and differences in compensatory movements according to motor impairment severity were not analyzed. Therefore, the observed increase in elbow flexion cannot be directly attributed to flexion synergy. Third, this study focused only on joint kinematics, and muscle activity was not assessed. Furthermore, the cross-sectional design does not allow causal inferences regarding the observed kinematic differences. Fourth, because maximal elevation differed between the paretic and non-paretic limbs, the same normalized points did not represent identical absolute angles. However, this normalization was used to identify when kinematic differences emerged during each limb's elevation. Despite these limitations, the present findings provide insight into when and how kinematic differences emerge during maximal upper-limb elevation in individuals with stroke.

## Conclusions

This study analyzed the compensatory movements of the shoulder, elbow, and trunk during maximal forward elevation of the upper limb in individuals with stroke. Changes in joint angles during early, middle, and maximal elevation were compared between the paretic and non-paretic limbs. The results showed no substantial postural differences between the two conditions up to middle elevation. However, at maximal elevation, increased elbow flexion was a prominent kinematic difference during elevation of the paretic limb. In contrast, shoulder abduction and internal rotation, movements that are often clinically considered compensatory, were not greater during paretic limb elevation than during non-paretic limb elevation. Rather, the shoulder internal rotation angle was significantly greater during non-paretic limb elevation than during paretic limb elevation. These findings suggest that increased elbow flexion may be an important kinematic feature to consider when evaluating maximal arm elevation in individuals with stroke, although further studies are needed to determine its clinical significance.

## References

[1] Thrane G, Alt Murphy M, Sunnerhagen KS (2018). Recovery of kinematic arm function in well-performing people with subacute stroke: a longitudinal cohort study. J Neuroeng Rehabil.

[2] Andrews AW, Bohannon RW (1989). Decreased shoulder range of motion on paretic side after stroke. Phys Ther.

[3] Rajaratnam BS, Venketasubramanian N, Kumar PV, Goh JC, Chan YH (2007). Predictability of simple clinical tests to identify shoulder pain after stroke. Arch Phys Med Rehabil.

[4] Zorowitz RD, Hughes MB, Idank D, Ikai T, Johnston MV (1996). Shoulder pain and subluxation after stroke: correlation or coincidence?. Am J Occup Ther.

[5] Bohannon RW, Larkin PA, Smith MB, Horton MG (1986). Shoulder pain in hemiplegia: statistical relationship with five variables. Arch Phys Med Rehabil.

[6] Dean CM, Mackey FH, Katrak P (2000). Examination of shoulder positioning after stroke: a randomised controlled pilot trial. Aust J Physiother.

[7] Jia F, Zhu XR, Kong LY (2023). Stiffness changes in internal rotation muscles of the shoulder and its influence on hemiplegic shoulder pain. Front Neurol.

[8] Schwarz A, Kanzler CM, Lambercy O, Luft AR, Veerbeek JM (2019). Systematic review on kinematic assessments of upper limb movements after stroke. Stroke.

[9] Gates DH, Walters LS, Cowley J, Wilken JM, Resnik L (2016). Range of motion requirements for upper-limb activities of daily living. Am J Occup Ther.

[10] Yamazaki S (1990). Fibrous structure of the joint capsule in the human shoulder. Okajimas Folia Anat Jpn.

[11] Meskers CG, Koppe PA, Konijnenbelt MH, Veeger DH, Janssen TW (2005). Kinematic alterations in the ipsilateral shoulder of patients with hemiplegia due to stroke. Am J Phys Med Rehabil.

[12] Thouant CL, Cocco ES, Morone G (2025). Compensation strategies in post-stroke individuals: insights from upper body kinematics analysis based on inertial sensors. Sensors (Basel).

[13] Takahashi K, Yamaji T, Wada N, Shirakura K, Watanabe H (2015). Trunk kinematics and muscle activities during arm elevation. J Orthop Sci.

[14] Brunnstrom S (1966). Motor testing procedures in hemiplegia: based on sequential recovery stages. Phys Ther.

[15] Sullivan KJ, Tilson JK, Cen SY (2011). Fugl-Meyer assessment of sensorimotor function after stroke: standardized training procedure for clinical practice and clinical trials. Stroke.

[16] Fugl-Meyer AR, Jääskö L, Leyman I, Olsson S, Steglind S (1975). The post-stroke hemiplegic patient. 1. a method for evaluation of physical performance. Scand J Rehabil Med.

[17] Mackey AH, Walt SE, Lobb GA, Stott NS (2005). Reliability of upper and lower limb three-dimensional kinematics in children with hemiplegia. Gait Posture.

[18] Robertson DGE, Dowling JJ (2003). Design and responses of butterworth and critically damped digital filters. J Electromyogr Kinesiol.

[19] Rab G, Petuskey K, Bagley A (2002). A method for determination of upper extremity kinematics. Gait Posture.

[20] Chénier F, Alberca I, Faupin A, Gagnon DH (2022). Interpreting the tilt-and-torsion method to express shoulder joint kinematics. Clin Biomech (Bristol).

[21] Ito D, Kawakami M, Hosoi Y, Kamimoto T, Yamada Y, Takemura R, Tsuji T (2024). Development of a quantitative assessment for abnormal flexor synergy index in patients with stroke: a validity and responsiveness study. J Neuroeng Rehabil.

[22] Aliaj K, Lawrence RL, Bo Foreman K, Chalmers PN, Henninger HB (2022). Kinematic coupling of the glenohumeral and scapulothoracic joints generates humeral axial rotation. J Biomech.

[23] Wagner JM, Dromerick AW, Sahrmann SA, Lang CE (2007). Upper extremity muscle activation during recovery of reaching in subjects with post-stroke hemiparesis. Clin Neurophysiol.

